# Evidence-Based Overview of Accelerometer-Measured Physical Activity during School Recess: An Updated Systematic Review

**DOI:** 10.3390/ijerph18020578

**Published:** 2021-01-12

**Authors:** Sergio Pulido Sánchez, Damián Iglesias Gallego

**Affiliations:** Physical Education & Exercise Lab, Teacher Training College, University of Extremadura, 10003 Cáceres, Spain; serpulsan@gmail.com

**Keywords:** physical activity, school recess, children, adolescents, systematic review

## Abstract

Interest in analyzing physically active behaviors during school recesses has grown in recent years as the school environment has consolidated (recess, physical education classes, lunch-time, before and after school) as a crucial space to bring these levels towards those recommended through intervention programs and improvements in the school environment. Unfortunately, in most of these studies, children do not achieve the 60 min a day of moderate to vigorous physical activity (MVPA) recommended by the World Health Organization. The aim of this systematic review is to analyze the cross-sectional, longitudinal, and intervention studies objectively measured with accelerometers that have emerged in recent years to determine the amount of MVPA of children at recess. This systematic review followed the PRISMA guidelines. The extraction process for the studies included in this systematic review yielded a total of 43 articles. The studies were classified according to the methodological nature of the research: cross-sectional (*n* = 34), longitudinal (*n* = 3) and quasi-experimental (*n* = 6). The results of the studies confirm that during the recess period younger children are physically more active than older ones and that in general, boys are more physically active than girls. In addition, the data show that the school contributes to more than 40% of the total MVPA. The intervention programs led to an increase in MVPA of up to 5%. Providing schools with equipment and facilities shows that intervention programs are beneficial for raising children’s levels of physical activity.

## 1. Introduction

The low levels of physical activity in the younger population are considered one of the main factors that cause health problems such as obesity or overweight, these levels being below the daily recommendations for moderate to vigorous physical activity [1]. In the United States, only about 42% of children between 6 and 11 years old meet the daily recommendations of 60 min a day, so it is necessary to study strategies to promote physical activity and health among children and adolescents [2]. Recent studies show that the environment affects the physical activity of children, since those who have large spaces, adequate equipment or a marking system in the schoolyard register higher levels of physical activity [3]. Children spend a very important proportional part of their daily work in the school environment, making school a great opportunity to influence levels of physical activity. However, each country is influenced by a series of important factors such as school policies, the programs that take place or the time allocated to recess [4]. Physical education classes, like recesses, are also periods that help students to reach the daily recommendations [5]. All these factors make physical activity levels vary from one country to another. In Japan, the proportion of moderate to vigorous physical activity performed by children is lower, around 18%, compared to European countries such as England or France where studies reported levels between 23.7% and 32.9% [3].

Most studies reveal that the levels of physical activity decrease with age, and that the physical activity of boys is higher than that of girls [6], since in general boys are more dominant in the yard during recess [7]. Other studies have also shown that there are no gender differences, although there are in the time spent in the different recess spaces, each one associated with different levels of physical activity [8]. There are many variables that influence the physical activity carried out by adolescents: the socioeconomic level of the parents, the intrinsic individual motivation and environmental factors such as the environment close to the subject, natural weather conditions or lack of time due to weather conditions, daily activities, and all of them can act in favor or to the detriment of the practice of physical activity [9]. In spite of everything, the opportunities offered by the school have improved in recent years and public health professionals have identified that schools, and specifically recess time, are a great setting for increasing the practice of physical activity by younger students [1]. Interventions in the schoolyard offer a good opportunity to improve students’ levels of physical activity [10]. Increasingly, children have the facilities to increase their daily physical activity at recess, this break time contributing around 40% of total activity and, in recent years, physical activity has been promoted in schools through different programs and strategies, such as marking the playground [11].

The use of accelerometry devices has allowed both physical activity professionals and the research community to extract quantifiable data on the real and objective amount of physical activity performed, and many studies use this instrument to objectively measure the physical activity in domains such as school [12]. With the help of emerging technology and research on this topic, an important challenge today is to try to take advantage of the spaces offered by the environment and the free time available to promote and encourage the active behaviors of those youngest [13].

The latest systematic reviews focused on the analysis of the literature on studies related to physical activity during school recess were carried out by Ridgers et al., in 2012 [4] and Parrish et al., in 2013 [11], for cross-sectional and intervention research, respectively. For this reason, the present systematic review took the indicated dates as its starting point, carrying out the search and analysis process until now (July 2020). More specifically, the objective of this study was to update the current state of knowledge about the determinants of active behavior during school recess. The systematic review adopted a three-fold approach to analysis, classifying the extracted studies according to their methodological nature: cross-sectional, longitudinal and quasi-experimental.

## 2. Methods

### 2.1. Search Strategy

This systematic review followed the PRISMA guidelines. The process of searching and the systematic review of the existing literature began in February 2020 and lasted approximately six months. The rigorous identification processes of the studies associated with the objective made it possible to group the most outstanding findings in a structured and organized way.

This systematic review of the scientific literature took as its starting point two previous ones carried out by Ridgers et al., in 2012 [4] and Parrish et al., in 2013 [11], corresponding to cross-sectional and intervention studies respectively. The reason that encouraged the realization of this work was the lack of updating in the large amount of scientific literature generated on the realization of physical activity during school recess. That is why for this documentary search, a screening of all the research articles between May 2011 and July 2020 was carried out for cross-sectional and longitudinal studies, and from April 2013 to July 2020 for quasi-experimental studies.

The Web of Science (WOS) database was mainly used for this literature review, although nine electronic literature databases were also involved in the process (Scopus, MedLine, SciELO, KCI, RSCI, Eric, PubMed, Dialnet and Google Scholar). The search strategies in the different databases were carried out by establishing descriptors (Table 1) in four large groups: population (child, children, youth, young, adolescent, student, students), school context (school, primary, elementary, middle school, high school, secondary school), recess time (break, breaks, recess, playtime, free play, free time), physical activity (physical activity). Only articles published in scientific journals were selected, excluding abstracts, books and conferences.

To guarantee the rigor of the research, a search was carried out with boolean markers using the following keywords [(“child” OR “children” OR “youth” OR “young” OR “adolescent” OR “student” OR “students”) AND (“School” OR “primary” OR “elementary” OR “middle school” OR “high school” OR “secondary school”) AND (“break” OR “breaks” OR “recess” OR “playtime” OR “free play” OR “free time”) AND (“physical activity” OR “physical activities”)]. In this way, it was possible to guarantee the non-exclusion of any results related to the research topic. It is important to note that regardless of the nature of the studies (cross-sectional, longitudinal or quasi-experimental) the keywords were the same for all search engines.

### 2.2. Inclusion/Exclusion Criteria

The inclusion criteria (Table 2) that were used to define the set of papers in this systematic review were the following: (1) Scientific journal publications in English; (2) Research published from the latest systematic reviews carried out; (3) Participants aged between 6 and 18 years; (4) Framed in the context of school recess; (5) Accelerometry as an instrument for measuring physical activity; (6) Contribution of values of physical activity of moderate to vigorous intensity (MVPA); (7) Exclusion of those intervention studies that do not include randomized controlled trials (RCT).

### 2.3. Data Extraction and Risk of Bias

The potentially eligible studies were initially screened by two reviewers (with previous experience in systematic reviews on physical activity in children and adolescents) independently by reading titles and abstracts, following the stipulated criteria prepared in advance, included in the search protocol [14]. In those studies with unclear abstracts or titles, a peer review was performed and the articles were agreed, resolving discrepancies through discussion and consensus [15]. In a second phase, the two reviewers independently read the full text of the studies preselected in the previous phase, creating the final list of potentially eligible studies, going to a third external investigator, when no consensus was reached regarding acceptability [16]. Finally, the full texts of the screened articles were carefully examined and analyzed.

## 3. Results

**Figure 1:**
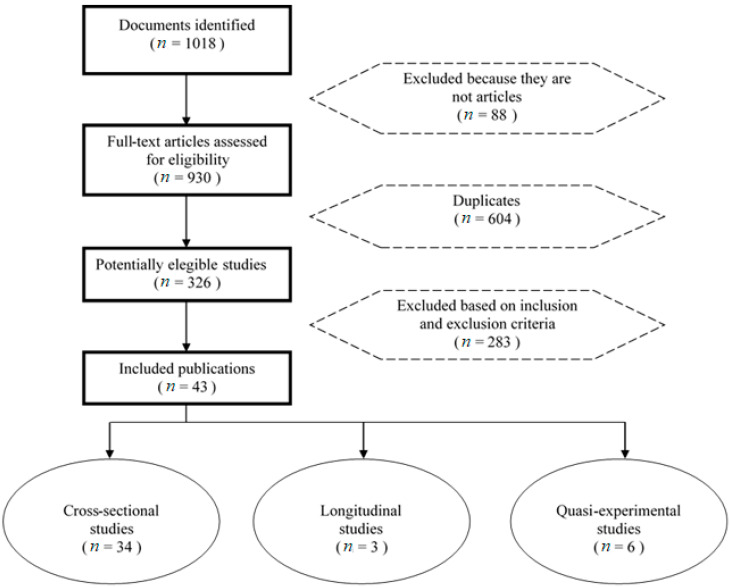
PRISMA flow diagram of systematic search and included studies.

This systematic review yielded a total of 1018 search results, of which 998 were within the time period delimited by the inclusion/exclusion criteria. The extraction process for the studies included in this systematic review yielded a total of 43 articles. The studies were classified according to the methodological nature of the research: cross-sectional (*n* = 34), longitudinal (*n* = 3) and quasi-experimental (*n* = 6). A summary of the PRISMA flowchart of each stage of the search can be seen in Figure 1.

Table 3 shows the main characteristics of the 34 cross-sectional studies found. A global analysis allows identifying a large set of variables related to this research topic (SED, PA, LPA, MPA, VHPA, MVPA, obesity, overweight, parental education level, ambulatory and non-ambulatory activity, ethnic groups), all of them conditioning the amount of physical activity that the subjects perform during recess. In general, younger children have higher levels of MVPA. It is also worth highlighting the evident difference that exists between sexes, since the levels of MVPA in boys are higher than those of girls. In some studies carried out by different authors in relation to the MVPA, girls performed 30.5%, while boys 47.0% [17]. Others studies found that the AFMV for boys was 17.9% and for girls 15.5% [4]. On the other hand, values of 30.1% versus 19.7%, respectively, were also found [18].

There were also significant effects in relation to programs and courses. Children’s MVPA at recess was higher than in physical education classes. In the lower grades, a lower sedentary behavior was demonstrated, accompanied by an increase in the MVPA of the first grade children during recess. The MVPA performed at recess is up to 39% higher than that performed in physical education classes [2] and the same happens in another study where PE only contributed to the 6.4% of the overall MVPA of children, while recess time contributed 18.7% and the results indicate that more than half of the children are reaching the daily recommendations and that recess and PE contribute to 43% of the total activity, so they are important moments for conducting MVPA [19].

**Table 3:** Main characteristics of cross-sectional studies.

Author/s (Year); Country [Reference]	Aims	Participants (Number of Schools, Age Range, Mean, Gender Distribution, Total)	Variable/s of PA	Individual, Social and Contextual Variables	Recess Duration and Description	Main Findings and Conclusions
Gao et al. (2015); United States [2]	Compare the different levels of low intensity PA in physical education, recess, and exercise programs.	*n* = 140 1 primary school 6–8 years 73 girls 67 boys	SED, LPA, MVPA (%)	Age, sex, grade, race	Recess duration is 20 min	Children’s MVPA is higher at recess than in physical education, up to 39%. Second graders are more sedentary than first graders during recess, and the younger they are, the higher their levels of MVPA.
Wood et al. (2014); United Kingdom [8]	The main objective was to examine the impact of the school play environment on children’s PA and to determine if the play environment influenced changes in self-esteem in the short term.	*n* = 25 1 urban primary school 8–9 years 13 girls 12 boys	MVPA (minutes)	Age, sex, height, weight, body mass index	Recess duration is 15 min	Boys are significantly more active than girls on the school playground, 4.8 ± 1.0 over 3.8 ± 0.9 min, but on the track there are no significant differences.
Wang (2019); China [20]	This study objectively measured the MVPA of children during recess and the different segments of the day.	*n* = 316 3 public elementary schools 6–13 years 144 girls 172 boys	MVPA (minutes)	Age, sex, grade, days of PE, days of EX	A long break of 30 min and 6 short breaks of 10 min each	Boys are significantly more active than girls during recess, performing 8.01 (6.6) minutes of PA on PE days compared to 6.1 (6.7) for girls. 2nd and 3rd grade students produce the most MVPA. The results recommend PA interventions during the less structured or unstructured segments.
Bailey et al. (2012); United Kingdom [17]	The objective of this study was to explore the compliance of boys and girls with the recommendations for MVPA during recess and other segments of the day.	*n* = 135 11 elementary schools10–14 years 78 girls 57 boys	SED, LPA, MPA, VPA, MVPA (%)	Age, race, height, weight, fat index, ACR	Recess lasts between 15 and 20 min.	Girls are significantly more sedentary than boys during recess. Boys’ PA is around 47% while that of girls is around 30.5%. PA patterns appear most beneficial to health in boys during less structured time periods and school interventions can help to seek opportunities for girls to be physically active during these times to overcome this observed sexual deficit.
Weaver et al. (2016); United States [21]	The purpose of this study was to identify the proportion of children who meet 30 min/day in MVPA and identify the segments of the school day in which the amount of MVPA varies for high and low activity children.	*n* = 323 7 schools 7.5 ± 1.1 173 girls 150 boys	MVPA (%)	Age, sex, grade, race, body mass index, PA level	Average recess time per day is 30.7 min	Most students do not accumulate 30 min per day of MVPA. The percentage of MVPA for boys is between 33.2 and 11.5 and for girls between 25.3 and 7.1. Physical education and class time are promising to promote MVPA.
Saint-Maurice et al. (2011); United States [22]	This study evaluated the usefulness of a multiple methods approach, accelerometers plus direct observation, in better understanding juvenile PA at recess.	*n* = 100 2 primary schools 8–12 years 48 girls 52 boys	MVPA (%)	Sex, grade, race, body mass index, seasons, type of activity	Recess lasts between 20 and 50 min	Comparisons between accelerometry and observation agree that boys are significantly more active than girls. In 3rd year 45.9% compared to 37.9%, in 4th year 34.1% compared to 30.1% and in 5th year 43.2% compared to 25.0%, differencing between boys and girls respectively. The MVPA increased in climbing activities and in those that were under supervision or with the use of equipment. If girls didn’t have to compete for play spaces it would be a great opportunity for them to be more active, but boys tend to occupy them.
Saint-Maurice et al. (2017); United States [23]	The purpose of this study was to empirically test the usefulness of specific calibration methods to allow the segmentation of PA levels in schoolchildren using established instruments.	*n* = 195 9 primary schools and 3 secondary schools	MVPA (%)	Age, sex, grade	Recess duration is 15.7 ± 7.5 min.	This study demonstrated that items in PAQ can be specified to predict MVPA minutes in youth groups during out-of-school periods. Recess is the second most active segment of the day and the results obtained from accelerometry and PAQ were similar, with a total of 20.4 ± 23.8 in the percentage of MVPA performed.
Viciana et al. (2016); Chile [24]	To compare the levels of MVPA objectively measured in PE and recess as well as to examine the influence of sex and weight.	*n* = 156 1 high school 13–14 years 69 girls 87 boys	MVPA (%)	Age, sex, weight, height, body mass index	Recess duration is 30 min	Statistics show that levels of MVPA vary in different contexts. Boys also do more PA than girls. Increasing PE hours and promoting PA at recess are good ways to increase PA levels for students in general and girls in particular. The total MVPA was around 6.94 ± 9.3.
Klinker et al. (2014); Denmark [25]	Employ new objective measures to assess age and gender differences in specific contexts and investigate associations between time spent outdoors and MVPA.	*n* = 170 4 schools 11–16 years	MVPA (minutes)	Age, sex, body mass index	Unspecified	Girls move less than boys, and children move more than adolescents. Context-specific patterns were found for gender and age, suggesting that different strategies may be necessary to promote PA, since the mean was approximately 5.5 min of MVPA.
Rooney et al. (2018); Ireland [19]	Objectives of this study were to objectively measure PA and establish the proportion of these children meeting current guidelines to determine to what extent PE and recess contribute to children’s overall PA, with a focus on age, sex and body composition.	*n* = 61 4 primary schools 8–11 years 37 girls 24 boys	SED, LPA, MPA, VPA, MVPA (%)	Age, sex, influence of PE	Recess duration is 15 min.	The students’ PA levels do not show significant changes the days that they do PE. PE only contributed 6.4% of the overall MVPA for children, while recess time contributed 18.7%. The results indicate that more than half of the children are reaching the daily recommendations and that recess and PE contribute 43% of the total activity, which is why they are important moments to produce MVPA.
Pollard et al. (2012); England [26]	The main aim of this study was to test if British Pakistani girls are less active than white British girls at recess.	*n* = 166 7 primary schools 9–11 years 70 white British 67 Pakistanis	SED, VPA, MVPA (%)	Age, grade, ethnicity, type of activity, behavior	Recess duration is 15 min	British Pakistani girls spend 2.2% less time in MVPA than white British girls. In the same way, they spend less time in the yard being active and playing. Recess is configured as a potential place to intervene and increase the PA they perform.
Ridgers et al. (2011); United States [27]	The purpose of this study was to examine PA levels during recess by sex, grade, ethnicity, and their contribution to daily PA.	*n* = 257 4 primary schools 8–12 years 139 girls 118 boys	SED, LPA, MPA, VPA, MVPA (%)	Age, height, weight, body mass index, degree of obesity and overweight, ethnicity.	Recess duration is 19.7 ± 6.5 min.	Boys are more active than girls during recess. No conclusive effects are observed according to ethnicity. Students in grades 3 and 5 are more active than in grades 4 and 6. Recess contributes to 17.9% and 15.5% for boys and girls respectively of the daily MVPA.
Kobel et al. (2015); Germany [28]	To investigate children’s PA levels during recess in German primary schools.	*n*=294 1 elementary school 7.1 ± 0.7 years 52% girls 48% boys	MVPA (minutes)	Age, sex, height, weight, body mass index and degree of obesity and overweight.	Recess duration is 30.65 ± 13.8 min	The students invested 25.8% of their recess in performing MVPA, with boys being more active than girls (30.28% over 20.4%). The time also differs significantly between students with a normal weight and with those who are overweight or obese, who perform less PA.
Galloway et al. (2019); United States [29]	Current policies on physical activity at school lack regulation, demonstrating the urgency of monitoring the MVPA, which is the purpose of this study.	*n* = 241 8 primary schools 9–10 years	SED, VPA, MVPA (minutes)	Age, sex, race, socioeconomic status	Recess duration is 29.4 ± 14.6 min	The results show an alarming situation regarding the lack of MVPA in the school. Girls are significantly less active than boys, 5.19 ± 3.03 versus 7.62 ± 5.87. Non-white children accumulate significantly more minutes of PA than white children.
Cohen et al. (2014); Australia [30]	The purpose of this study was to examine the associations between the fundamental principles of the movement and MVPA objectively measured in the school day among children attending elementary schools in low-income communities.	*n* = 460 8 primary schools 8.5 ± 0.6 years 54% girls 46% boys	MVPA (minutes)	Age, sex, body mass index, socioeconomic status, motor skills.	Recess lasts approximately 20 min.	Motor skills competence was positively associated with MVPA and as a good way to increase PA levels, particularly in skills related to object control. Boys accumulated 7.0 ± 4.0 min of MVPA and girls 4.8 ± 4.1 min.
Tanaka et al. (2019); Japan [18]	The main objective of the study is to describe PA levels during school and recess and to compare them between both sexes.	*n* = 409 14 urban primary schools 6–12 years 223 girls 186 boys	SED, LPA, MPA, VPA, MVPA (%)	Age, sex, height, weight, body mass index, degree of overweight and obesity, ambulatory and non-ambulatory activity.	Recess duration is between 15–20 min.	Boys perform significantly more PA than girls, 30.1% versus 19.7%. These findings suggest that the total amount of time spent at recess or lunch probably contributes to the daily MVPA, but the beneficial effects should be explored in future intervention studies.
Béghin et al. (2019); Different countries of the European Union [9]	Compare the differences in PA performed between the participants according to their age and sex.	*n* = 1842 12.5–17.4 years	MVPA (minutes)	Age, sex, height, weight, body mass index, nutritional status, parental education level, smokers, puberty level	Unspecified	There are no significant differences between the PMP and PP groups in relation to MVPA for girls. However, PMP children are much more active than PP children during recess, 7.4 ± 1.4 min versus 6.3 ± 1.4.
Massey et al. (2018); United States [31]	The purpose of this study was to study the individual variables associated with PA performed by children during recess, as well as environmental influences.	*n* = 146 7 primary schools 9.85 years on average	MVPA (minutes)	Age, sex, race, economic level of the school, need for psychological satisfaction, autonomy, skills, relationship.	Recess duration is between 20–30 min.	Recess time and sex differences are significant indicators of PA during school recess. The commitment and supervision of adults is also a positive indicator in the performance of PA of children. The MVPA of boys is higher than that of girls, 10.09 min compared to 7.10 min.
Ridgers et al. (2014); United States [32]	Investigate children’s PA levels during recess and their contribution to daily PA in relation to their weight status.	*n* = 217 4 primary schools 7–12 years 118 girls 99 boys	SED, LPA, MPA, VPA, MVPA (%)	Age, sex, height, weight, body mass index, degree of overweight	Recess has an average duration of 19.7 ± 6.5 min.	There are no significant differences for MVPA between overweight and non-overweight students, although there are notable differences in relation to MPA and VPA, and also between boys (49.5%) and girls (34.8%).
Baquet et al. (2014); France [33]	The objective of this study was to objectively measure the schoolchildren PA at recess and relate it to socioeconomic levels.	*n* = 407 4 primary schools 6–11 years 201 girls 206 boys	SED, LPA, MPA, VPA, VHPA, VPA + VHPA, MVPA (%)	Age, gender, SES	Recreation between 1300 and 1500 m^2^ 2 low SES schools and two high SES schools.	Boys are significantly more active than girls. Students with a high SES present higher levels of PA than those with a low SES, 30.1% compared to 26.1%. This study indicates that interventions in recess should have low SES schools as the objective of action.
Andersen et al. (2015); Denmark [34]	This study investigated the use of different school areas during recess and its relationship with PA.	*n* = 316 4 schools 10–15 years 168 girls 148 boys	SED, MVPA (%)	Age, sex, grade, playgrounds.	The average duration of recess is 25.6 min. It is divided into five areas: natural, grass court, lawn, pavement, recreation area.	The grass court and recreation area are associated with higher PA levels. Boys accumulate more PA time than girls, 4.6% out of 3.0%, and boys in general more than adolescents. It is important to research the playgrounds to promote the practice of PA in school.
McWhannell et al. (2019); United Kingdom [35]	Compare the PA levels of two schools with different SES.	*n* = 53 2 primary schools 7–8 years 27 girls 26 boys	SED, LPA, MPA, VPA, MVPA (%)	Age, gender, SES	Recess duration is 15 min.	Boys and girls with higher SES spend more time in MVPA. The MVPA increases significantly in children with high SES over those with low SES by up to 12.4% more, while in girls the changes are not so remarkable, with only a 1.8% increase.
Hubáckovà et al. (2016); Poland [6]	The main objective was to establish differences between the levels of PA and inactivity of boys and girls in primary and secondary schools on the different segments of the day.	*n* = 395 9 primary schools and 12 secondary schools 9–17 years 184 girls 211 boys	MVPA (minutes per hour)	Age, sex, height, weight, body mass index, grade.	Unspecified	Boys and girls in primary school are more active than in secondary school. Boys are more active than girls for both types of schools. Primary school children perform 22.08 min of MVPA per hour while secondary school children 3.93 min.
Pan et al. (2015); United States [36]	Compare the PA levels of high school students with and without autism and check which ones meet the daily recommendations.	*n* = 60 22 secondary schools 12–17 years 30 with autism and 30 without autism	MPA, VPA, MVPA (%)	Age, sex, autism	Recess duration is 10 ± 1.37 min. 6 breaks.	The PA for children with autism is significantly lower. Schools should increase opportunities for PA throughout the day.
Silva et al. (2015); Portugal [13]	Examine children’s PA levels during free periods from school and their contribution to daily PA.	*n* = 213 1 urban public school and 1 rural public school 14 ± 1.7 years 135 girls 78 boys	SED, MVPA (minutes)	Age, sex, weight, height, body mass index, type of school.	Recess duration is 60 min.	Boys’ PA is significantly higher than girls. Children in the rural school are more active than in the urban school. The contribution of PA for both sexes was 37.7–42.7% for girls and 40.1–42.1% for boys.
Vanhelst et al. (2017); European Cities [37]	Establish relationships between school rhythms and adolescent PA.	*n* = 2024 12.5–17.5 years 1015 girls 1008 boys	SED, MVPA (minutes)	Age, sex, height, weight, body mass index, weight status, puberty status, parental education	Recess duration is 40–90 min depending on school rhythms.	The students in the group with long rhythms performed higher MVPA than those with short rhythms, 9.8 ± 7.9 versus 3.9 ± 4.0. PA recommendations were also poorer in the short rhythm group, 30.7% vs. 34.1% of long rhythms. The results suggest taking advantage of school time to promote PA.
Martin et al. (2012); Australia [38]	The objective of this study was to investigate the characteristics associated with the school environment in relation to MVPA at recess.	*n* = 408 27 metropolitan elementary schools11 years 187 girls 221 boys	MVPA (minutes)	Age, sex, weight, race, school policies, teachers, school environment, SES.	Recess duration is 60 min.	About 40% of the variability of children in relation to the MVPA is determined by the school environment and the individual characteristics identified in this study. Greater MVPA is associated with new schools, better coordination, and more field areas. School and environmental characteristics are closely related to the amount of PA performed.
Van Kann et al. (2016); Holland [39]	Investigate the relationship between recess and MVPA and SED in 8–11 year old students.	*n* = 257 20 public schools 8–11 years 137 girls 120 boys	SED, MVPA (minutes)	Age, sex, grade, school policies, school environment.	One break in the morning of 11.3 ± 3.3 and another in the afternoon of 25.5 ± 15.3 min.	On average, students spend 54 min in the playground, of which 9 are from MVPA. Boys perform higher MVPA than girls, 2.43 ± 2.21 vs. 1.55 ± 1.60 in the morning recess and 5.88 ± 4.53 vs. 3.96 ± 4.11 in the afternoon. The use of equipment such as bars and goals is associated with higher levels of PA.
Dessing et al. (2013); Holland [40]	Examine the time and intensity of the students’ PA in the different segments of the day.	*n* = 76 6 primary schools 6–11 years 44 girls 32 boys	MVPA (minutes)	Age, sex, height, weight, body mass index, school location.	Unspecified	On average, schoolchildren spend 40.1 min a day on the playground. In school recess, boys are more active than girls with 39.5% out of 23.4% of MVPA. This time contributes 17.5% and 16.8% to the daily recommendations of boys and girls respectively.
Nettlefold et al. (2011); Canada [41]	Describe PA through the study of the school day and the daily recommendations for boys and girls.	*n* = 379 9 primary schools 8–11 years 197 girls 182 boys	SED, LPA, MVPA (minutes, %)	Age, sex, height, weight, body mass index.	Recess lasts between 15 and 25 min.	Girls accumulate 1.6 fewer minutes of MVPA than boys at recess. Only some boys and girls reach the daily recommendations, 34.1% and 15.7% respectively. Schools should complement PE with models that increase opportunities to do PA at any time of the day.
Klinker et al. (2014); Denmark [12]	Identify the domains and subdomains in MVPA objectively measured between sexes and ages.	*n* = 367 4 schools 13.2 ± 1.2 years 192 girls 175 boys	MVPA (minutes, %)	Age, sex, grade, body mass index, parental employment, ethnicity.	Unspecified	Boys (15.8%) accumulate a greater amount of MVPA than girls (10.7%) and the same happens with children (14.8%) compared to adolescents (9.8%).
Suzuki et al. (2018); Japan [42]	Examine students’ PA patterns and identify their periods of influence.	*n* = 40 5 primary schools 10.00 ± 1.51 years 18 girls 22 boys	SED, LPA, MPA, VPA, MVPA (minutes, %)	Age, sex, grade, body mass index.	Recess duration is 15–20 min.	MVPA was higher before school and at recess (<15%) than after school (<10%). Students who spent their time on the playground achieved higher levels of MVPA. The activities carried out in school have a great influence on the PA of school children.
Woods et al. (2018); United States [43]	Investigate the behaviors, actions and attitudes of elementary school children who are considered not very active in relation to those who are more active.	*n* = 179 4 elementary schools 85 girls 94 boys	SED, LPA, MPA, VPA, MVPA (%)	Age, sex, grade, group.	Each recess period lasts between 12 and 25 min.	The participants of the not very active sample performed MVPA for 17.5% of recess, while the total sample is framed around 34.5%, choosing mainly individual activities (57.7%). Less active students attribute it to an increase in social interactions over the performance of PA.
Viciana et al. (2019); Chile [44]	To examine the objectively measured differences between PA and SED in adolescents.	*n* = 156 1 municipal school 13.41 years on average 69 girls 87 boys	LPA, MVPA (%)	Age, sex, weight, height, body mass index, degree of obesity or overweight.	Three breaks of 10 and 30 min up to a total duration of 60 min.	Recess is the best moment in the realization of MVPA. The MVPA of boys (9.39%) is significantly higher than that of girls (2.56%).

*n* = sample; SED: sedentary behavior; PA: physical activity; LPA: light physical activity; MPA: moderate physical activity; VHPA: very high physical activity; MVPA: moderate to vigorous physical activity; ST: staff training; EQ: equipment; d = effect size (Cohen); F = effect of the intervention; PE = physical education; EX = physical exercise; ACR = cardiorespiratory attitude; PMP = pre/mid puberty; PP = post puberty; SES: socioeconomic status.

Table 4 shows the main characteristics of the 3 longitudinal studies found. More specifically, a study on a marking program in the schoolyard, another on physical activity in the different seasons of the year and finally another that relates physical activity to recess and lunchtime. In general, over time, benefits are achieved in terms of increased physical activity. The marking system in the patio or the improvement of the patio areas favors the practice of physical activity, with significant changes just six months after the start [45]. Regarding the seasons of the year, the data reveal that the physical activity carried out decreases in the summer months (19.5%) and spring (24.4%), with the highest levels being in winter (27.7%) [46]. The analysis of the data also allows us to observe that girls are more active in dance activities, a low court or a climbing structure, while boys are more active in an obstacle course [47]. As can be seen, the duration of recess time varies from one study to another depending on the country where they were carried out, being 30 min in a single period in some studies [46] and 60 min distributed in four periods in other studies [47].

Table 5 shows the main characteristics of the six intervention studies found. Two of the studies are based on the “Ready for recess” program, two on the “Playworks” program, one on a marking system in play areas and the last one on changes in the playing environment. Thanks to these programs, five of the six studies corroborate the importance of designing recess programs to increase the practice of physical activity, in which increases in physical activity carried out by students of up to 19.4% are observed. However, one of the studies, despite indicating that these interventions are positive, does not show a significant increase in the physical activity of the sample in the accelerometry devices [48].

According to “Ready for Recess” program, boys in FP + EQ increased their MVPA by 14.1% while girls remain slightly more sedentary after the intervention [49]. In another study, a significant increase in MVPA was also observed in the experimental group compared to the control group [7]. Furthermore, teachers in schools that made use of the “Playworks” program reported an increase in levels of physical activity, but accelerometry measures and student surveys did not show significant impacts [48]. Nevertheless, future studies should redirect their analysis in such a way that they can explore the underlying mechanisms in the intervention proposals that are carried out in schools [7].

**Table 5:** Main characteristics of quasi-experimental studies.

Author/s (Year); Country [Reference]	Aims	Participants (Number of Schools, Age Range, Mean, Gender Distribution, Total)	Variable/s of PA	Individual, Social, Contextual Variables	Recess Duration and Description	Characteristics of the Intervention	Main Findings	Conclusions
Blaes et al. (2013); France [10]	Improve PA at recess through intervention with markings in the playing area	*n* = 332 4 primary schools 6–11 years 165 girls 167 boys	SED, LPA, MPA, VPA, MVPA (%)	Age (years), weight (kg), height (cm), body mass index (kg m^−2^), body mass index (%)	1300 to 1500 m^2^ of recreation between asphalt and green areas Brands in the yard worth €15,000 Two 15 min recesses	Design of the playground by zones with three specific areas of play. The red one (sports), the blue one (multiple activities) and the yellow one (fast zone)	At the beginning, the PA of the CG is slightly higher than that of the EG. After the intervention, the PA of the EG (27 ± 9.9) increased slightly while that of the CG (29.5 ± 11.1) hardly varied.	Painting markings in the play area has positive effects on students’ PA levels at recess
Huberty et al. (2014); United States [49]	Determine Ready for Recess effectiveness, a program of intervention aimed at staff training or providing recreational equipment, separately, and the combination of the 2 on the time of boys and girls in MVPA during recess.	12 primary schools 7–12 years 559 girls 523 boys	SED, MVPA (%)	Grade (range), gender (n,%), body mass index, age (years)	The time students spend at recess is 21 ± 6.3 min	Materials were used to plan the activities in each zone and included cones, zone markers and play equipment. The equipment was made available depending on the space of the area.	Boys in FP + EQ increased their MVPA by 14.1% while girls remain slightly more sedentary after the intervention, going from 24.0 ± 5.4 to 23.5 ± 4.3 of MVPA.	Environmental modifications are only as strong as the personnel who implement them. Supervision, if not interactive, can be detrimental to PA, especially of girls.
Huberty et al. (2011); United States [50]	Determine effectiveness Ready for Recess, a program of intervention aimed at staff training or providing recreational equipment, separately, and the combination of the 2 on the time of boys and girls in MVPA during recess.	*n* = 262 4 primary schools 141 girls 121 boys	MVPA (%)	Grade (range), gender (n,%), race, body mass index	The time students spend at recess is 19.8 ± 7.4 min	Materials were used to plan the activities in each zone and included cones, zone markers and play equipment. The equipment was made available depending on the space of the area.	Boys in ST + EQ increased their MVPA by 19.4% and girls by 6%.	Ready for Recess represents a possible means of increasing MVPA in obese and overweight students, that is, in populations less likely to meet MVPA recommendations.
Bleeker et al. (2015); United States [51]	The aim of this article is to investigate the effects of Playworks on girls and boys and physical activity separately using data collected using accelerometers and structured recess observations.	*n* = 1573 29 primary schools 823 girls 750 boys	MVPA (%)	Gender	The measurement was made during at least 10 min of recess. The recess was divided into different observation areas.	The person in charge of carrying out Playworks in each school tries to involve students in physical activity by providing equipment, such as balls and cones, introducing organized games within different areas or designated zones on the playground.	Girls who participate in the Playworks program are more active than those who do not participate, going from 27.5% to 33.3%. A significant impact was found on the types of activities that girls participated in during recess.	Playworks had a significant impact on girls’ PA measurements, however it was not as significant in boys.
Beyler et al. (2014); United States [48]	Evaluate the impact of Playworks on students’ PA during recess.	*n* = 157329 primary schools	VPA, MVPA (%)	Age, gender, race	The measurement was made during at least 10 min of recess. The recess was divided into different observation areas.	During recess, the Playworks coach engages students in physical activity by encouraging their participation in organized and inclusive activities such as Four Squares, Simon Says, Fronton, and Basketball, as well as giving them rules and guidelines for resolving conflicts.	A significantly higher percentage of teachers reported that their students participated in intense PA during recess. No significant differences (from 16.4% to 21.4%) were found for student reports on their physical activity during recess.	Teachers at Playworks schools reported that students were more active during recess, but accelerometer measurements and student surveys did not show marginally significant impacts.
Yildirim et al. (2014) Unspecified [7]	Increase the PA of children and decrease your sedentary behavior through a environmental intervention at school and familiar surroundings	*n* = 268 20 primary schools 57% girls 43% boys 8.2 years	LPA, MVPA (%)	Age (years), Gender (% girls), weight (kg), body mass index (kg m-2), body mass index (kg/m^2^), state of overweight and obesity (%), parental education level (%)	31 ± 5 recess minutes	Intervention in four groups: intervention in sedentary behaviors, intervention in PA behaviors, the previous two combined and a control group, through perceived social support from teachers, scoreboards, sports equipment and the school perceived as a play environment.	There was a significant effect in the intervention group compared to the control group in relation to the MVPA, which mainly had a positive effect in girls. The MVPA evolves from 22% to 29%.	A positive perception of the school environment was associated with a higher MVPA during recess in girls. Future studies should conduct mediation analyzes to explore the underlying mechanisms of PA interventions.

*n* = sample; SED: sedentary; PA: physical activity; LPA: light physical activity; MPA: moderate physical activity; VHPA: very high physical activity; MVPA: moderate to vigorous physical activity; EG: experimental group; CG: control group; ST: staff training; EQ: equipment.

## 4. Discussion

The aim of this systematic review was to investigate the determining factors of active behavior during school recess, analyzing the levels of physical activity performed by children in this time frame and their impact on the global values of daily physical activity of the school-age population. Although the results of these studies show heterogeneity and not all of them provide significant evidence on the potential of the school as an institution that promotes physical activity, we can observe that most of the investigations agree that recess is a key moment to promote the levels of physical activity towards the recommended 60 min of daily MVPA. Other variables of physical activity that are reflected on in this research topic are light physical activity (LPA), moderate physical activity (MPA), vigorous physical activity (VPA) and sedentary behaviors (SED), expressed in minutes or in percentage, always in relation to the 60 min recommended by the World Health Organization and that guarantee objective measures that allow knowing real data about what is happening in schoolyards.

The realization of this update of the existing literature has had a triple approach, collecting cross-sectional, longitudinal and quasi-experimental studies, which implies an important strength of this systematic review, focused specifically on physical activity during school recess. The sample of students from the different schools has presented a great variety from some studies to others, ranging between 25 and 2024 subjects. Of the studies analyzed, 14 of them have been carried out in the United States, with the vast majority of the rest coming mainly from countries of the European Union.

From a global point of view we can appreciate the wide range of variables that are collected in the scientific literature and how each of them influences in relation to the levels of physical activity measured objectively with accelerometry, most of them being individual variables that directly affect the active behavior of the subject, such as age and gender. First, boys engage in more physical activity than girls, probably due to their nature, since boys tend to spend most of their time doing physical activities and competing with each other, while girls spend their time mostly in social activities [17,18]. In addition, it is recorded in numerous studies that boys tend to occupy the vast majority of play spaces and that the violence of their activities relegates girls to being located in the corners of the patio [4]. Second, we find that age is also a limiting factor. Younger children are generally significantly more active than older children. This is because as many of them grow up they lose interest and motivation in the practice of physical activity and that day-to-day activities considerably reduce the time they have for it [41]. That is why the need arises to design intervention programs that encourage physical activity both within the school environment and outside.

Although the objective of this study is to examine the amount of physical activity that children perform in relation to MVPA, we can find other variables that have been included by other studies presented here and that may be related with levels of physical activity at recess (such as the socioeconomic status of students or disorders such as obesity and autism).Children from families with a higher socioeconomic level perform more MVPA than those who live in families with a low socioeconomic level [30]. Other students who find their physical condition limited by the different disorders that are collected in the studies (whether they refer to eating disorders or others such as autism or the loss of some sense) also present lower levels of MVPA than those students exempt from them. The need arises to devise programs to adapt the centers to the different needs of their students and to create habits in students that allow them to develop physical activities both inside and outside of school [29].

The duration of recess is also a variable that we can find in all studies. In some schools, the recess time is divided into the morning recess and the lunch time recess, being typical of the schools that have a split day. Other studies are carried out in schools with up to four recess periods, which positively favors the performance of physical activity by students, since more than 40% of the children’s MVPA is carried out at school. The time for recess for the sample of articles is between 10 and 60 min.

Regarding the intervention studies, it should be noted that all of them correspond to studies carried out under the RCT premise so that their results are not biased by manipulation of the groups and the results are obtained by a randomized controlled trial. The six quasi-experimental studies analyzed in this review sought to increase the physical activity of students through intervention programs or improvements in the school environment (i.e., “Ready for recess” program, “Playworks” program, “marking system in play areas”). It is clear that teachers and school policies can have a significant impact on the physical activity carried out by children during school recesses. Most of the strategies reported benefits for students regardless of gender. The work of teachers is necessary to carry out these programs to prevent boys from dominating the entire play space and to compensate for the difference that exists with girls in increasing levels of physical activity. Some strategies such as the implementation of markings in the playground, the remodeling of the play areas or the implementation of play equipment are configured as potential strategies to increase the levels of physical activity in the school environment, but there is a need to continue working on it. The findings currently found are on the right track.

## 5. Conclusions

School recess is a space to potentially improve levels of daily physical activity in the lives of children. Gender and age are determinants, as at all levels girls are significantly less active than boys and prone to develop sedentary behaviors. As they grow, levels of physical activity also decreased, the smallest people performing the greatest MVPA. There are other variables that act positively or negatively related to physical activity levels present in children. Socioeconomic status is a variable to be highlighted because children with higher SES spend more time in MVPA. There is a need to advocate for interventions at school to promote moderate to vigorous physical activity (i.e., “Ready for recess” program, “Playworks” program, “marking system in play areas”). Environmental changes are as strong as the staff implements. Supervision is crucial because if it is not interactive, it can be detrimental to the PA, especially girls. Teachers should pay special attention to schedules to have more control of the variables of physical activity.

In conclusion, the school context is configured as a key space to meet the daily recommendations for physical activity. Studies show that by improving the conditions of the courtyard, providing it with equipment equipped according to the needs of students, improve play areas and designing programs that promote physical activity are to achieve positive improvements in levels of MVPA. For future research, it would be very useful for the scientific community to continue studying the relationship between the variables of physical activity and school recess, specifically in quasi-experimental studies, in which the capacity of these intervention programs to increase the levels of physical activity up to those recommended is analyzed, since most of the articles are cross-sectional in nature but there are very few longitudinal and intervention ones, in which this need for RCTs should be qualified as a criterion for the research design.

## Figures and Tables

**Table 1:** Systematic review categories and search terms.

Population	School Context	Recess Time	Physical Activity
child children youth young adolescent student students	school primary elementary middle school high school secondary school	break breaks recess playtime free play free time	physical activity physical activities

**Table 2:** Inclusion/exclusion criteria.

Category	Description	Comments
Type of document	Scientific articles from English language journals	No abstracts, books or conference proceedings
Time period	2011–2020 (cross-sectional and longitudinal) 2013–2020 (intervention)	The most recent reviews are taken as starting point
Educational level	Primary Education Secondary Education High School Education	6–18 years old
Timing	School recess	Not specific to recess
Types of studies	Only articles that objectively measure physical activity variables with accelerometry	Excluding all items that do not use this instrument
Main variable	MVPA	At least include this variable
RCT	Interventions should be randomized controlled trials	Excluded non-randomized group studies

MVPA: moderate to vigorous physical activity; RCT: randomized controlled trials.

**Table 4:** Main characteristics of longitudinal studies.

Author/s (Year); Country [Reference]	Aims	Participants (Number of Schools, Age Range, Mean, Gender Distribution, Total)	Variable/s of PA	Individual, Social, Contextual Variables	Recess Duration and Description	Mainfindings and Conclusions
Baquet et al. (2018); France [45]	The purpose of this study was to monitor the effects of an intervention through markings in school recess to measure children’s PA levels over 12 months and to highlight factors associated with sedentary behavior and different PA levels.	*n* = 326 3 primary schools 6–11 years 162 girls 164 boys	SED, LPA MVPA (%)	Age, sex, height, weight, body mass index, time	Recreation space between 1300–1500 m^2^ Two 15-min recesses	After 6 months of intervention in the playground, there is an increase in LPA and after 12 months the MVPA generally improved 3.36% in the youngest, assuming 5.44% more MVPA for children. This study shows that intervening through markings on the playground has positive effects on students’ MVPA. However, in 12 months there are no changes in relation to the LPA and SED.
Ridgers et al. (2018); Australia [46]	The objective of this study was to determine how children’s physical activity levels influence during recess and lunchtime and if their contribution to daily physical activity differed according to the seasons.	*n* = 326 9 primary schools 8–11 years 164 girls 162 boys	LPA, MPA, VPA, MVPA (%)	Age, height, weight, temperature, humidity, wind speed, seasons of the year	Recess duration is 29.7 ± 2.7 min	The MVPA during lunchtime decreases significantly in spring 24.4 (13.1) and summer 19.5 (10.7) compared to winter 27.7 (12.4). However, during recess no significant changes are observed.
Andersen et al. (2019); Denmark [47]	The aim of this work was to investigate the time spent and PA levels in three renovated paved schoolyards in Denmark, with a special interest in finding activation areas and facilities with high levels of physical activity.	*n* = 323 3 primary schools 11–15 years	SED, LPA, MVPA (minutes)	Age, sex, year of follow-up	Recess duration is approximately 60 min distributed between 2 to 4 periods. Schoolyard renovation cost between $586,000 $670,000 Balance beams, small hills, dance areas, skating areas, multiple courts, and Panna-Court rinks (small circular play area with two goals designed for games like soccer or hockey)	Evidence-based solutions for schoolyard design can help in an effort to provide good opportunities for children to be more physically active during recess. Courtyards with large asphalt areas offer good opportunities for children. It is suggested from the study that the schoolyards have multifunctional areas such as courts, marking areas or dance areas. Ball courts or marking zones generated AF points for both sexes. Girls are most active in dance activities, a low court, or a climbing frame, while boys are on an obstacle course. In the first school it was 9.2 (6.8), in the second it was 10.1 (6.4) and for the third it was 14.9 (8.0) minutes of MVPA.

*n* = sample; SED: sedentary behavior; PA: physical activity; LPA: light physical activity; MPA: moderate physical activity; VHPA: very high physical activity; MVPA: moderate to vigorous physical activity; ST: staff training; EQ: equipment.

## References

[1] Tercedor P., Segura-Jiménez V., Ávila García M., Huertas-Delgado F.J. (2019). Physical activity during school recess: A missed opportunity to be active?. Health Educ. J..

[2] Gao Z., Chen S., Stodden D.F. (2015). A comparison of children’s physical activity levels in physical education, recess, and exergaming. J. Phys. Act. Health.

[3] Ishii K., Shibata A., Sato M., Oka K. (2014). Recess physical activity and perceived school environment among elementary school children. Int. J. Environ. Res. Public Health.

[4] Ridgers N.D., Salmon J., Parrish A.M., Stanley R.M., Okely A.D. (2012). Physical activity during school recess: A systematic review. Am. J. Prev. Med..

[5] Grao-Cruces A., Segura-Jiménez V., Conde-Caveda J., García-Cervantes L., Martínez-Gómez D., Keating X.D., Castro-Piñero J. (2019). The Role of School in Helping Children and Adolescents Reach the Physical Activity Recommendations: The UP&DOWN Study. J. Sch. Health.

[6] Hubáčková R., Groffik D., Skrzypnik L., Frömel K. (2016). Physical activity and inactivity in primary and secondary school boys’ and girls’ daily program. Acta Gymnica.

[7] Yildirim M., Arundell L., Cerin E., Carson V., Brown H., Crawford D., Hesketh K.D., Ridgers N.D., TeVelde S.J., Chinapaw M.J.M. (2014). What helps children to move more at school recess and lunchtime? Mid-Intervention results from Transform-Us! Cluster-randomised controlled trial. Br. J. Sports Med..

[8] Wood C., Gladwell V., Barton J. (2014). A repeated measures experiment of school playing environment to increase physical activity and enhance self-esteem in UK school children. PLoS ONE.

[9] Béghin L., Vanhelst J., Drumez E., Migueles J.H., Androutsos O., Widhalm K., Julian C., Moreno L.A., De Henauw S., Gottrand F. (2019). Gender influences physical activity changes during adolescence: The HELENA study. Clin. Nutr..

[10] Blaes A., Ridgers N.D., Aucouturier J., Van Praagh E., Berthoin S., Baquet G. (2013). Effects of a playground marking intervention on school recess physical activity in French children. Prev. Med..

[11] Parrish A.M., Okely A.D., Stanley R.M., Ridgers N.D. (2013). The effect of school recess interventions on physical activity: A systematic review. Sports Med..

[12] Klinker C.D., Schipperijn J., Christian H., Kerr J., Ersbøll A.K., Troelsen J. (2014). Using accelerometers and global positioning system devices to assess gender and age differences in children’s school, transport, leisure and home based physical activity. Int. J. Behav. Nutr. Phys. Act..

[13] Silva P., Sousa M., Sá C., Ribeiro J., Mota J. (2015). Physical activity in high school during ‘free-time’ periods. Eur. Phys. Educ. Rev..

[14] Viswanathan M., Patnode C.D., Berkman N.D., Bass E.B., Chang S., Hartling L., Murad M.H., Treadwell J.R., Kane R.L. (2018). Recommendations for assessing the risk of bias in systematic reviews of health-care interventions. J. Clin. Epidemiol..

[15] Higgins J.P.T., López-López J.A., Becker B.J., Davies S.R., Dawson S., Grimshaw J.M., McGuinness L.A., Moore T.H.M., Rehfuess E.A., Thomas J. (2019). Synthesising quantitative evidence in systematic reviews of complex health interventions. BMJ Glob. Health.

[16] Van Sluijs E.M., McMinn A.M., Griffin S.J. (2007). Effectiveness of interventions to promote physical activity in children and adolescents: Systematic review of controlled trials. BMJ.

[17] Bailey D.P., Fairclough S.J., Savory L.A., Denton S.J., Pang D., Deane C.S., Kerr C.J. (2012). Accelerometry-assessed sedentary behaviour and physical activity levels during the segmented school day in 10–14-year-old children: The HAPPY study. Eur. J. Pediatr..

[18] Tanaka C., Tanaka M., Inoue S., Okuda M., Tanaka S. (2019). Gender differences in physical activity and sedentary behavior of Japanese primary school children during school cleaning time, morning recess and lunch recess. BMC Public Health.

[19] Rooney L., McKee D. (2018). Contribution of physical education and recess towards the overall physical activity of 8–11 year old children. J. Sport Health Res..

[20] Wang L. (2019). Accelerometer-determined physical activity of children during segmented school days: The Shanghai perspective. Eur. Phys. Educ. Rev..

[21] Weaver R.G., Crimarco A., Brusseau T.A., Webster C.A., Burns R.D., Hannon J.C. (2016). Accelerometry-Derived Physical Activity of First Through Third Grade Children During the Segmented School Day. J. Sch. Health.

[22] Saint-Maurice P.F., Welk G.J., Silva P., Siahpush M., Huberty J. (2011). Assessing children’s physical activity behaviors at recess: A multi-method approach. Pediatr. Exerc. Sci..

[23] Saint-Maurice P.F., Welk G.J., Bartee R.T., Heelan K. (2017). Calibration of context-specific survey items to assess youth physical activity behaviour. J. Sports Sci..

[24] Viciana J., Mayorga-Vega D., Martínez-Baena A. (2016). Moderate-to-vigorous physical activity levels in physical education, school recess, and after-school time: Influence of gender, age, and weight status. J. Phys. Act. Health.

[25] Klinker C.D., Schipperijn J., Kerr J., Ersbøll A.K., Troelsen J. (2014). Context-specific outdoor time and physical activity among school-children across gender and age: Using accelerometers and GPS to advance methods. Front. Public Health.

[26] Pollard T.M., Hornby-Turner Y.C., Ghurbhurrun A., Ridgers N.D. (2012). Differences between 9–11 year old British Pakistani and White British girls in physical activity and behavior during school recess. BMC Public Health.

[27] Ridgers N.D., Saint-Maurice P.F., Welk G., Siahpush M., Huberty J.L. (2011). Differences in Physical Activity duringSchool Recess. J. Sch. Health.

[28] Kobel S., Kettner S., Erkelenz N., Kesztyüs D., Steinacker J.M. (2015). Does a Higher Incidence of Break Times in Primary Schools Result in Children Being More Physically Active?. J. Sch. Health.

[29] Galloway R., Booker R., Owens S. (2019). Factors Leading to Discrepancies in Accumulated Physical Activity during School Hours in Elementary School Students. J. Teach. Phys. Educ..

[30] Cohen K.E., Morgan P.J., Plotnikoff R.C., Callister R., Lubans D.R. (2014). Fundamental movement skills and physical activity among children living in low-income communities: A cross-sectional study. Int. J. Behav. Nutr. Phys. Act..

[31] Massey W.V., Stellino M.B., Fraser M. (2018). Individual and environmental correlates of school-based recess engagement. Prev. Med. Rep..

[32] Ridgers N.D., Saint-Maurice P.F., Welk G.J., Siahpush M., Huberty J.L. (2014). Non-overweight and overweight children’s physical activity during school recess. Health Educ. J..

[33] Baquet G., Ridgers N.D., Blaes A., Aucouturier J., Van Praagh E., Berthoin S. (2014). Objectively assessed recess physical activity in girls and boys from high and low socioeconomic backgrounds. BMC Public Health.

[34] Andersen H.B., Klinker C.D., Toftager M., Pawlowski C.S., Schipperijn J. (2015). Objectively measured differences in physical activity in five types of schoolyard area. Landsc. Urban Plan..

[35] McWhannell N., Triggs C., Moss S. (2019). Perceptions and measurement of playtime physical activity in English primary school children: The influence of socioeconomic status. Eur. Phys. Educ. Rev..

[36] Pan C.Y., Hsu P.J., Chung I.C., Hung C.S., Liu Y.J., Lo S.Y. (2015). Physical activity during the segmented school day in adolescents with and without autism spectrum disorders. Res. Autism Spectr. Disord..

[37] Vanhelst J., Béghin L., Duhamel A., De Henauw S., Molnar D., Vicente-Rodriguez G., Manios Y., Widhalm K., Kersting M., Polito A. (2017). Relationship between school rhythm and physical activity in adolescents: The HELENA study. J. Sports Sci..

[38] Martin K., Bremner A., Salmon J., Rosenberg M., Giles-Corti B. (2012). School and individual-level characteristics are associated with children’s moderate to vigorous-intensity physical activity during school recess. Aust. N. Z. J. Public Health.

[39] Van Kann D.H.H., de Vries S.I., Schipperijn J., de Vries N.K., Jansen M.W.J., Kremers S.P.J. (2016). Schoolyard Characteristics, Physical Activity, and Sedentary Behavior: Combining GPS and Accelerometry. J. Sch. Health.

[40] Dessing D., Pierik F.H., Sterkenburg R.P., van Dommelen P., Maas J., de Vries S.I. (2013). Schoolyard physical activity of 6–11 year old children assessed by GPS and accelerometry. Int. J. Behav. Nutr. Phys. Act..

[41] Nettlefold L., McKay H.A., Warburton D.E.R., McGuire K.A., Bredin S.S.D., Naylor P.J. (2011). The challenge of low physical activity during the school day: At recess, lunch and in physical education. Br. J. Sports Med..

[42] Suzuki I., Okuda M., Tanaka M., Inoue S., Tanaka S., Tanaka C. (2018). Variability in school children’s activity occurs in the recess and before-school periods. Pediatr. Int..

[43] Woods A.M., McLoughlin G.M., Kern B.D., Graber K.C. (2018). What’s Physical Activity Got to Do with It? Social Trends in Less Active Students at Recess. J. Sch. Health.

[44] Viciana J., Mayorga-Vega D., Parra-Saldías M. (2019). Within and between-day differences in adolescents’ objectively-measured physical activity and sedentary behavior. Kinesiology.

[45] Baquet G., Aucouturier J., Gamelin F.X., Berthoin S. (2018). Longitudinal Follow-Up of Physical Activity during School Recess: Impact of Playground Markings. Front. Public Health.

[46] Ridgers N.D., Salmon J., Timperio A. (2018). Seasonal changes in physical activity during school recess and lunchtime among Australian children. J. Sports Sci..

[47] Andersen H.B., Christiansen L.B., Pawlowski C.S., Schipperijn J. (2019). What we build makes a difference—Mapping activating schoolyard features after renewal using GIS, GPS and accelerometers. Landsc. Urban Plan..

[48] Beyler N., Bleeker M., James-Burdumy S., Fortson J., Benjamin M. (2014). The impact of Playworks on students’ physical activity during recess: Findings from a randomized controlled trial. Prev. Med..

[49] Huberty J.L., Beets M.W., Beighle A., Saint-Maurice P.F., Welk G. (2014). Effects of ready for recess, an environmental intervention, on physical activity in third-through sixth-grade children. J. Phys. Act. Health.

[50] Huberty J.L., Beets M.W., Beighle A., Welk G. (2011). Environmental modifications to increase physical activity during recess: Preliminary findings from ready for recess. J. Phys. Act. Health.

[51] Bleeker M., Beyler N., James-Burdumy S., Fortson J. (2015). The Impact of Playworks on Boys’ and Girls’ Physical Activity during Recess. J. Sch. Health.

